# Development and Mechanistic Evaluation of Polymeric Nanomicrogels Under High-Temperature and High-Salinity Conditions

**DOI:** 10.3390/gels11090689

**Published:** 2025-08-30

**Authors:** Wei Zhang, Yinbo He, Tengfei Dong, Huayan Mu, Guancheng Jiang, Quande Wang

**Affiliations:** 1 College of Petroleum Engineering, China University of Petroleum (Beijing), Beijing 102249, China; 17653991333@163.com (W.Z.); heyb@cup.edu.cn (Y.H.); 15664628289@163.com (Q.W.); 2 College of Science, China University of Petroleum (Beijing), Beijing 102249, China; dtf010019@163.com; 3 College of Artificial Intelligence, China University of Petroleum (Beijing), Beijing 102249, China; a_corresponding@163.com

**Keywords:** deep/ultra-deep reservoirs, ultra-high-temperature resistance, salt resistance, microgel, lost circulation control

## Abstract

Fracture-induced loss poses severe challenges to drilling operations, particularly under high-temperature and high-salinity conditions encountered in deep wells. Conventional plugging materials, characterized by relatively large particle sizes and poor structural integrity, often exhibit insufficient thermal stability and salt tolerance under extreme drilling conditions, making them prone to structural degradation and loss of adhesion, which ultimately leads to drilling fluid deterioration and downhole complications. To address this issue, a core–shell-structured microgel, ANDT-70 (named after the acronyms of 2-acrylamido-2-methylpropane sulfonic acid, N-vinyl-2-pyrrolidinone, N, N-dimethylacrylamide, dimethyl diallyl ammonium chloride, and titanium dioxide nanoparticles), was synthesized and systematically evaluated for its thermal stability, salt resistance, and interfacial adhesion capabilities. The structural evolution, dispersion behavior, and colloidal stability of the microgel were thoroughly characterized using scanning electron microscopy (SEM), transmission electron microscopy (TEM), atomic force microscopy (AFM), Raman spectroscopy, and Zeta potential analysis. Experimental results indicate that ANDT-70 exhibits excellent thermal stability and resistance to salt-induced degradation at 260 °C, maintaining its fundamental structure and performance under harsh high-temperature and high-salinity conditions, with a viscosity retention of 81.10% compared with ambient conditions. Compared to representative materials reported in the literature, ANDT-70 exhibited superior tolerance to ionic erosion in saline conditions. AFM analysis confirmed that ANDT-70 significantly improves bentonite slurry dispersion and reduces salt sensitivity risks. ANDT-70 stably adsorbs onto bentonite lamellae via the synergistic action of electrostatic interactions and hydrogen bonding, thereby forming a dense cementation network that markedly enhances the structural stability and adhesion of the system. This network significantly enhances the cohesion and structural integrity of drilling fluid systems under extreme conditions. In conclusion, ANDT-70 demonstrates strong potential as a high-performance functional microgel for enhancing the stability and effectiveness of advanced drilling fluids under complex geological environments.

## 1. Introduction

Fracture-induced losses have long posed significant challenges in petroleum engineering. With the depletion of shallow resources and advancements in deep drilling technologies, well depths now often exceed 5000 m, creating high-temperature and high-salinity conditions that place stringent demands on drilling fluid performance. These extreme environments not only accelerate drilling fluid degradation and resource loss, but also substantially increase the risk of severe downhole incidents, such as wellbore collapse and blowouts [1,2,3,4]. Statistics indicate that fracture-induced loss results in annual eco-nomic damage of up to USD 4 billion and account for approximately 10% of non-productive time [1]. Traditional granular loss control materials are often too large to effectively seal loss channels ranging from tens of microns to several millimeters [5,6]. In addition, they typically lack adequate thermal stability and salt tolerance, which leads to performance degradation under harsh conditions. Polymer chains tend to coil in saline high-temperature environments, deactivating functional groups and reducing sealing efficacy. These issues can lead to significant problems such as drilling fluid failure, resulting in significant losses, wellbore instability, and reservoir damage. Therefore, it is of great significance to design and develop cementing materials with excellent adhesion properties to address such environments and achieve efficient and stable oil and gas production in high-temperature high-salt wells [7,8,9].

Most current plugging gels are derived from acrylamide (AM) or acrylic acid (AA), which offer good swelling and gelation properties [10,11]. However, these materials predominantly rely on bulk expansion to seal pores, which limits their effectiveness in sealing microfractures due to their relatively large particle sizes [12,13]. Natural additives such as xanthan gum have been introduced to improve gel elasticity, but these materials typically suffer from thermal degradation and structural failure in high-temperature high-salinity environments, severely compromising their sealing capacity and potentially triggering fluid loss and wellbore instability [14,15,16,17]. To enhance the adaptability of drilling fluids under extreme conditions, researchers have turned to the synergistic integration of nanomaterials and polymer systems. Nanomaterials, with their high surface area, excellent dispersibility, and thermal stability, significantly improve the interfacial performance and structural integrity of gel systems. Numerous studies have demonstrated that incorporating nano-particles into gels enhances their thermal and salt resistance, improves rheological properties, and strengthens interfacial adhesion [18,19,20].

In recent years, various nanomaterials have been widely utilized to enhance the performance of water-based drilling fluids, demonstrating significant potential [21,22]. Nanomaterials, with their excellent dispersibility and stability, can enhance dispersion and adhesion in systems and reduce fluid loss. The introduction of nanoparticles also enhances thermal stability and adaptability [23,24]. Yang Liu et al. [25] reported that nanomaterials are suitable for addition to gels as plugging materials and compared the performance of gels containing titanium dioxide and silicon dioxide. Their results indicated that nano-TiO_2_ is more suitable for addition to gels to prepare high-temperature plugging materials. Feiyang Huang et al. [26] prepared a high-temperature thixotropic polymer nanogel with a three-dimensional crosslinked network structure from a composite material of nanolapasite and the thermosensitive polymer poly (acrylamide-acrylamidemorpholine-2-acrylamide-2-methylpropanesulfonic acid) (P(AM-ACMO-AMPS)), which exhibits excellent plugging performance and corrosion resistance. Buddhabhushan Salunkhe et al. [27] developed a unique hydrogel with ultra-high-temperature tolerance (HT-PPG). HT-PPG can swell to over 30 times its initial volume in saline solutions of varying ionic strengths, making it an ideal candidate for applications under harsh temperature and salinity conditions. Reem Elaf, Ahmed Ben Ali et al. [28] developed a green biodegradable polyacrylamide gel (PAM/Cs) containing poly-acrylamide and chitosan as a water-plugging agent. PAM/Cs exhibited excellent swelling and rheological properties under high-temperature and high-salinity conditions, as well as long-term thermal and hydrolytic stability. Bowen Yu et al. [29] systematically evaluated a modified PPG product, high-temperature-resistant, re-crosslinkable, and pre-formed particle gel (HT-RPPG). This gel can re-crosslink within macroporous features after injection to form a volume-expanding material while maintaining thermal stability. Yunhai Zhao et al. [30] synthesized a crosslinked polyethyleneimine polymer and introduced nanoparticles to create a silica nanoparticle-coordinated crosslinked polymer system. The nanosynergistic BAtBA/PEI crosslinked polymer system exhibits excellent stability and selective plugging ability under high-temperature and high-salinity conditions, showing promising applications in the petroleum industry. Dehghani et al. [31] synthesized a multifunctional nanomaterial that exhibits excellent viscosity-modulating properties under high salinity by integrating multiple functional nano-particles (e.g., Al_2_O_3_, MgO, SiO_2_, and CaCO_3_).

Although nanomaterial-enhanced gels have shown promising improvements in thermal resistance, salt tolerance, and sealing efficiency, the long-term stability and adhesion mechanisms of nanomicrogels under harsh conditions such as high temperature and high salinity remain insufficiently understood. In this study, a nanocomposite microgel, ANDT-70, was synthesized for high-temperature and high-salinity drilling conditions. Its thermal stability, salt resistance, and interfacial adhesion capabilities were systematically evaluated through structural characterization and performance testing. Techniques including scanning electron microscopy (SEM), transmission electron microscopy (TEM), atomic force microscopy (AFM), Raman spectroscopy, and Zeta potential analysis were employed to comprehensively reveal the morphological evolution, dispersion behavior, and colloidal stability of the microgels, thereby clarifying the relationship between their structure and performance. Results confirmed that ANDT-70 maintains a stable three-dimensional structure and interfacial functionality under harsh conditions, demonstrating excellent thermal stability and resistance to salt-induced degradation. This study provides a theoretical foundation and technical guidance for the application of high-performance nanomicrogels in extreme drilling environments.

## 2. Results and Discussion

### 2.1. Characterization of ANDT-70

**Figure 1:**
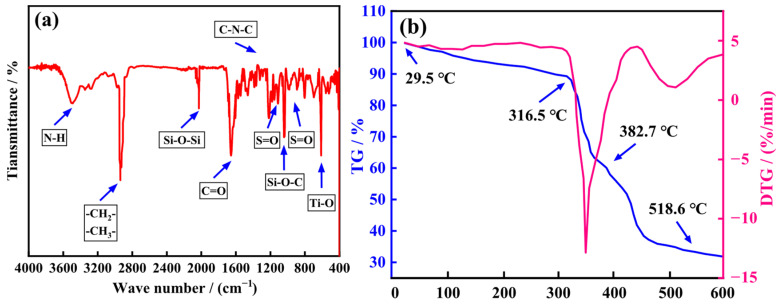
The FTIR (**a**) and TG (**b**) curves of acrylic resin.

The Fourier transform infrared (FTIR) spectrum of the core–shell-structured microgel ANDT-70 is shown in Figure 1a. Sharp peaks at 817.5 cm^−1^ and 1089.1 cm^−1^ correspond to the characteristic Si-O-C vibrations, while the sharp peak at 2026 cm^−1^ is attributed to the Si-O-Si stretching vibration, representing the characteristic peaks of TiO_2_ and 3-Methacryloxypropyltrimethoxysilane (KH570), respectively. The sharp peaks at 1037.03 cm^−1^ and 3423.99 cm^−1^ are assigned to the N-H and S=O stretching vibrations of the 2-acrylamido-2-methylpropane sulfonic acid (AMPS) units, respectively. The sharp peaks observed in the range of 2840–2950 cm^−1^ are associated with the -CH_2_ and -CH_3_ stretching vibrations. A sharp peak at 1637.36 cm^−1^ corresponds to the C=O stretching vibration of tertiary amide groups present in N-vinyl-2-pyrrolidinone (NVP) and N, N-dimethylacrylamide (DMAA) units. The sharp peak at 1363.96 cm^−1^ arises from the C-N-C stretching vibration of the imide structure in the NVP units. Therefore, the FTIR results confirm the presence of the designed functional groups in the core–shell microgel ANDT-70, consistent with the intended molecular structure.

If a microgel is used as a plugging agent but lacks sufficient thermal resistance, its performance may deteriorate due to thermal degradation, resulting in reduced plugging efficiency. Therefore, it is crucial to investigate the thermal tolerance of the core–shell-structured microgel ANDT-70 developed in this study. As shown in Figure 1b, the thermal degradation process occurs in three distinct stages. The first stage takes place below 316.5 °C and is characterized by a gradual mass loss, primarily attributed to the evaporation of free and bound water. The second stage occurs between 316.5 °C and 382.7 °C, where a significant mass loss is observed due to the thermal decomposition of sulfonic acid groups, amide groups, and other thermally labile functional moieties. The third stage, ranging from 382.7 °C to 600 °C, involves further mass loss attributed to structural rearrangement and the cleavage of the microgel polymer backbone. The results indicate that ANDT-70 exhibits excellent thermal stability below 316.5 °C, demonstrating its significant potential for application in deep-well and high-temperature environments.

### 2.2. Thermal Stability Analysis

**Figure 2:**
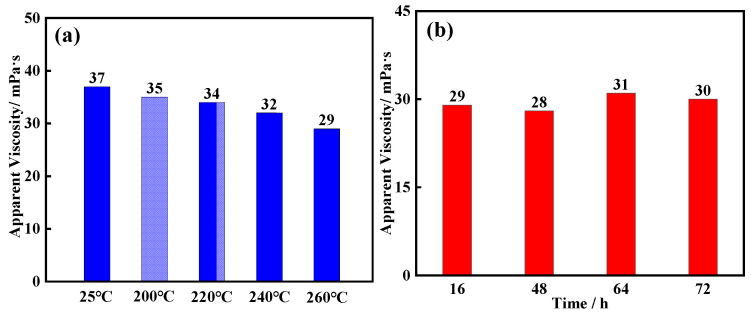
Thermal stability evaluation: temperature tolerance (**a**), long-term thermal resistance (**b**).

Following the procedure described in Section 4.4, temperature tolerance and long-term thermal stability tests were conducted on a bentonite slurry system modified with the core–shell-structured microgel ANDT-70. The results are shown in Figure 2. As shown in Figure 2a, the viscosity of the system decreases with increasing temperature. However, after aging at 260 °C for 16 h, the system maintains a viscosity of approximately 29 mPa·s, indicating that the high temperature imposes minimal impact on the system, and no significant structural degradation has occurred. Even after continuous hot rolling for 72 h under high-temperature conditions, the viscosity remains relatively stable, maintaining around 29 mPa·s, as illustrated in Figure 2b. Compared to room-temperature conditions, the viscosity retention rate was 81.10%. These findings demonstrate that the core–shell-structured microgel ANDT-70 exhibits excellent thermal tolerance and long-term heat resistance, indicating strong potential for application in extreme high-temperature and high-pressure downhole environments.

**Figure 3:**
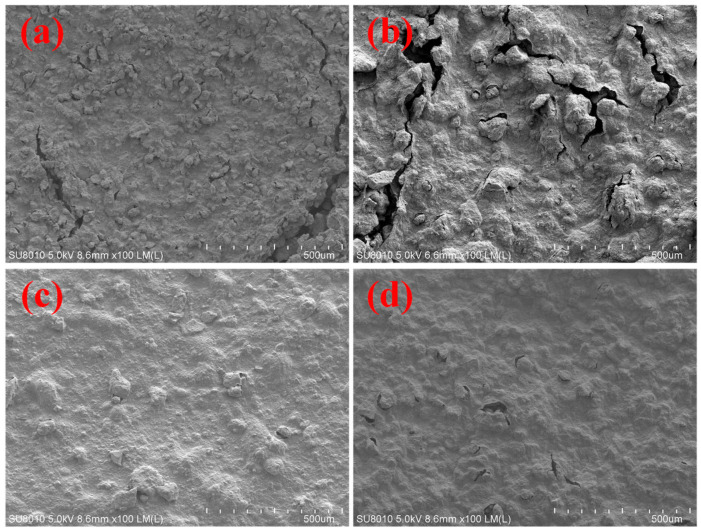
SEM test results: SEM of bentonite slurry before aging (**a**), SEM of bentonite slurry after aging (**b**), SEM before aging with the addition of ANDT-70 (**c**), SEM after aging with the addition of ANDT-70 (**d**).

The microstructure of bentonite slurry, both before and after the incorporation of core–shell-structured microgel ANDT-70, was thoroughly investigated using scanning electron microscopy (SEM) to elucidate its mechanism in enhancing high-temperature and long-term thermal resistance. As shown in the SEM images, compared to the bentonite slurry under ambient conditions (Figure 3a), the sample subjected to aging at 260 °C for 16 h (Figure 3b) exhibited numerous surface cracks, a noticeably rough texture, and the formation of larger aggregates. This phenomenon is likely attributed to the thermal disruption of the layered structure of bentonite, resulting in structural instability and particle aggregation. The presence of cracks and aggregates significantly reduced the adhesive strength of the system and impaired the cementing efficiency of bentonite within rock pores. Figure 3c,d reveal that the incorporation of ANDT-70 microgel notably improved the microstructure of the system. The surface appeared relatively smooth and uniform, with no evident aggregation or structural collapse. The preserved compact structure is considered a key factor contributing to the enhanced adhesion performance of the system. The SEM results further confirm that the ANDT-70 microgel significantly enhances the thermal tolerance and long-term thermal stability of the bentonite slurry system.

**Figure 4:**
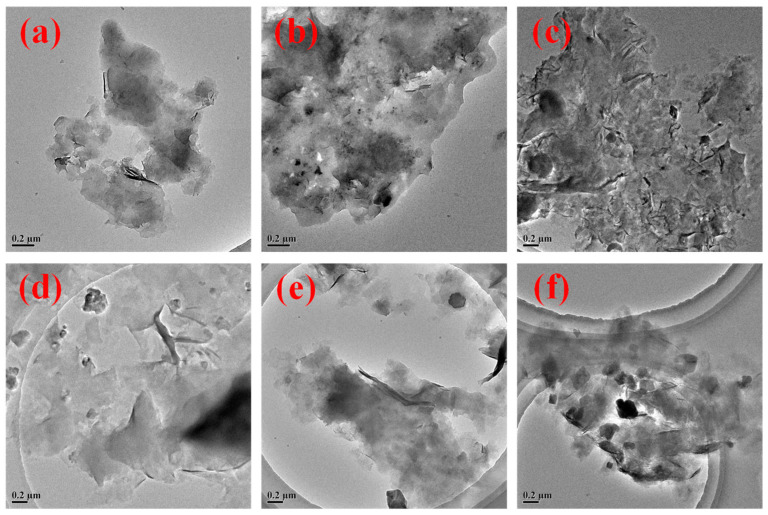
TEM test results: TEM of bentonite slurry before aging (**a**), TEM of bentonite slurry after aging (**b**), TEM of bentonite slurry after aging with the addition of salt ions (**c**), TEM before aging with the addition of ANDT-70 (**d**), TEM after aging with the addition of ANDT-70 (**e**), TEM after aging with the addition of salt ions and ANDT-70 (**f**).

To further investigate the high-temperature performance mechanism of the core–shell-structured microgel ANDT-70, transmission electron microscopy (TEM) was employed to observe the microstructures of bentonite slurry with and without the addition of ANDT-70. As shown in the TEM images, the bentonite slurry exhibited reduced transparency after high-temperature treatment, accompanied by the pronounced aggregation of bentonite lamellae (Figure 4a,b). In contrast, the addition of ANDT-70 significantly enhanced the transparency of the system, indicating that the microgel effectively inhibited the aggregation of bentonite lamellae (Figure 4d,e), which is critical for maintaining high thermal resistance. Additionally, salt ions were introduced into the system to evaluate the effects of salt-induced erosion. A comparison of Figure 4c,f reveals that the introduction of ANDT-70 substantially reduced particle aggregation and effectively protected the bentonite structure from salt-ion-induced degradation. Further validation of the salt resistance of ANDT-70 is provided in Section 2.3 in detail.

The findings from high-temperature resistance and long-term thermal stability tests, corroborated by TEM and SEM microstructural analyses, consistently demonstrate that the incorporation of ANDT-70 significantly enhances the thermal tolerance and structural integrity of the system.

### 2.3. Salt Resistance Analysis

**Figure 5:**
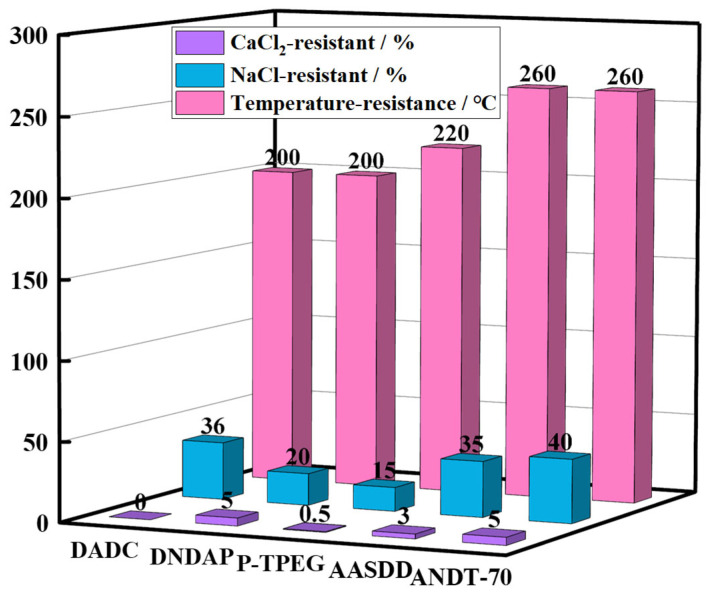
Comparison of salt ion tolerance performance of microgels ANDT-70.

To further evaluate the salt resistance of the core–shell-structured microgel ANDT-70, a comparative analysis was conducted against representative salt-tolerant materials reported in the literature [19,32,33,34], as shown in Figure 5. To ensure fair comparison, the experiments were conducted under the same conditions as reported in the literature and carried out following the procedures described in Section 4.4 of this study. In saturated NaCl systems, the performance of ANDT-70 was similar to that of AASDD, exhibiting superior salt resistance over other materials. In calcium salt environments, ANDT-70 demonstrated a performance similar to that of DNDAP and outperformed the remaining reference materials. In summary, considering the findings in Section 2.2 regarding thermal endurance and long-term stability, ANDT-70 exhibits robust overall stability and application potential under high-temperature, prolonged exposure, and high-salinity conditions.

**Figure 6:**
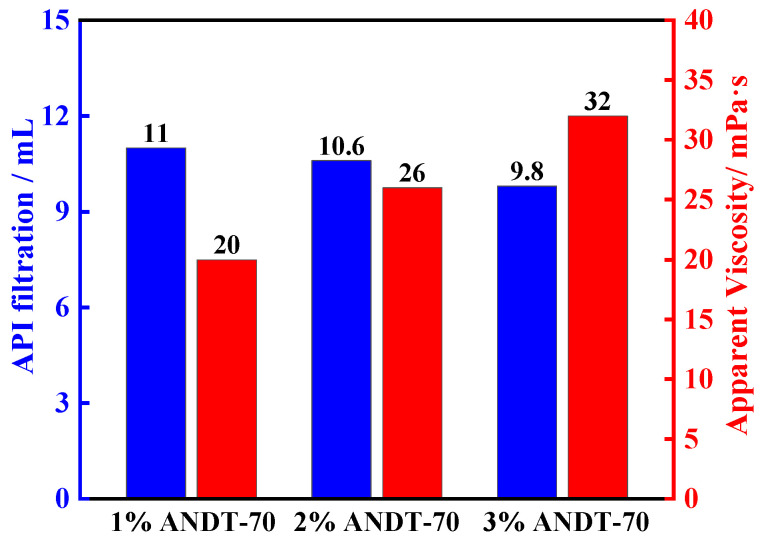
Performance test results of bentonite slurry under different concentrations of ANDT-70.

**Figure 7:**
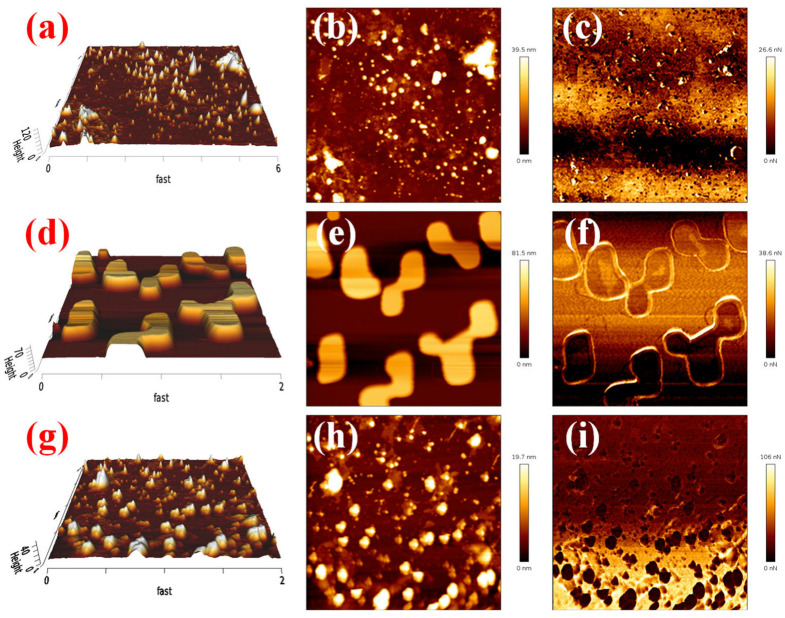
AFM test results: AFM image of bentonite slurry (**a**–**c**), AFM image with the addition of salt ions (**d**–**f**), AFM image with the addition of ANDT-70 (**g**–**i**).

To investigate the relationship between ANDT-70 and drilling fluid performance parameters (American Petroleum Institute (API filtration) and rheology), the performance of bentonite slurry drilling fluids was evaluated at varying ANDT-70 concentrations. The results show that the system viscosity increased with higher ANDT-70 content (Figure 6). Taking into account that excessively high viscosity can negatively affect drilling fluid performance, 2 wt% ANDT-70 was found to achieve optimal fluid properties. To investigate the mechanism by which the core–shell-structured microgel ANDT-70 enhances the salt ion resistance of bentonite slurry, three dispersions were prepared: bentonite slurry with salt ions and ANDT-70, bentonite slurry alone, and bentonite slurry with salt ions. Each sample was diluted to 10^−6^ for analysis and to ensure that the instrument operates within its measurement range, thereby enhancing methodological transparency and data reliability. The microstructures of these samples were examined using atomic force microscopy (AFM). As shown in Figure 7a,b, the bentonite slurry dispersion exhibited good dispersibility with an average particle height of 39.5 nm. Upon the addition of salt ions, significant aggregation and layer stacking occurred. The average height increased to 81.5 nm (Figure 7c), indicating that salt ion erosion disrupted the bentonite structure and promoted aggregate formation. This suggests that the bentonite slurry system is prone to structural destabilization and aggregation failure under high salinity conditions. However, with the addition of ANDT-70, the average height significantly decreased to 19.7 nm (Figure 7h), indicating improved dispersion and a notable reduction in aggregation risk (Figure 7g). These AFM results confirm that the microgel ANDT-70 effectively enhances the resistance of the bentonite slurry system to salt ion erosion.

### 2.4. Bonding Adhesion Analysis

**Figure 8:**
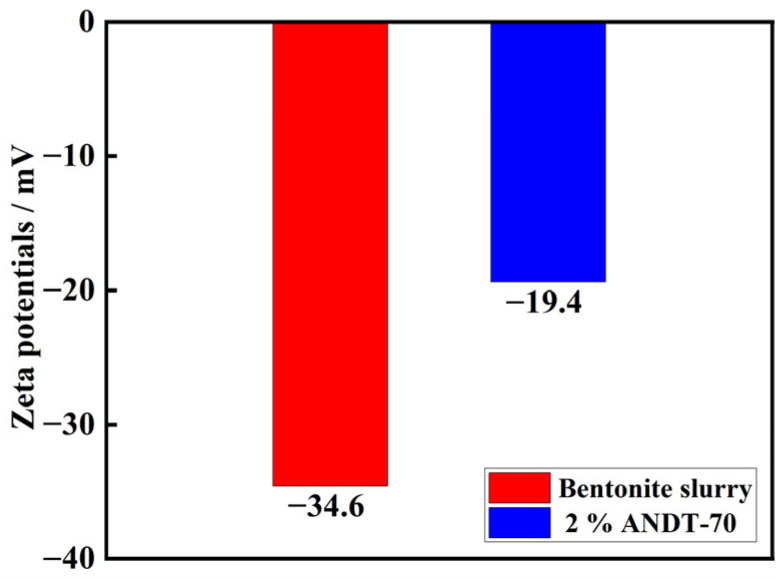
Zeta potential test results.

To investigate the mechanism by which the core–shell-structured microgel ANDT-70 affects the adhesion properties of the bentonite slurry system, the Zeta potential of bentonite slurry dispersions before and after ANDT-70 addition was measured. As shown in Figure 8, the Zeta potential of the bentonite slurry dispersion was −34.6 mV, which increased to −19.4 mV upon the addition of microgel ANDT-70. This change indicates that ANDT-70 reduces the negative surface charge density of the system through electrostatic interactions. Moreover, ANDT-70 chemically adsorbs onto the surface of bentonite slurry lamellae and strengthens interlayer adhesion via electrostatic forces and hydrogen bonding. Furthermore, the improved electrostatic stability facilitates the formation and maintenance of the cohesive gel network.

**Figure 9:**
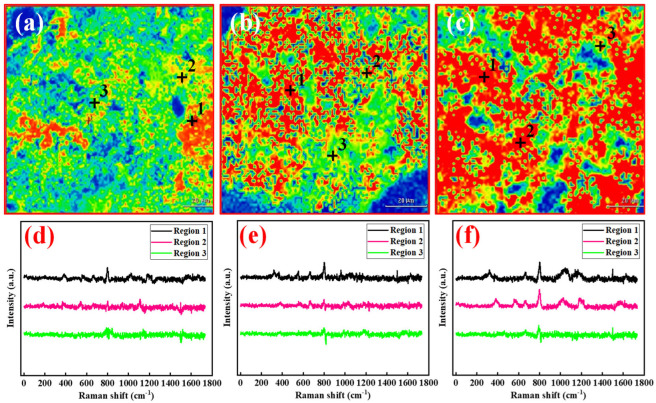
Raman testing of the surface of slices after core displacement experiments with 0.5 wt% concentration of ANDT-70 (**a**,**d**), Raman testing of the surface of slices after core displacement experiments with 1.0 wt% concentration of ANDT-70 (**b**,**e**), Raman testing of the surface of slices after core displacement experiments with 2.0 wt% concentration of ANDT-70 (**c**,**f**), (Three test locations were randomly selected for analysis and marked on the image).

Raman spectroscopy and Raman mapping techniques were employed to investigate the effect of varying concentrations of microgel ANDT-70 on the cementation ability of bentonite slurry systems. Bentonite slurries containing 0.5 wt%, 1.0 wt%, and 2.0 wt% ANDT-70 were prepared, followed by core flooding and subsequent Raman analysis. At 0.5 wt% concentration, fewer Raman peaks were observed on the core cross-section, indicating limited interaction of ANDT-70. The corresponding Raman mapping (Figure 9a) showed sparse ANDT-70 distribution, further confirming its limited contribution to cementation. As the concentration increased, Raman signals intensified, and Raman mappings (Figure 9b,c) exhibited increasingly dense ANDT-70 distribution, indicating enhanced surface adsorption and cementation. At 2.0 wt%, the red regions in the Raman map significantly expanded, dominating the area (Figure 9c), and distinct Raman peaks were observed (Figure 9f), demonstrating a pronounced enhancement in cementation and adhesion performance (The darker the Raman imaging color, the denser the distribution of ANDT-70, and the stronger the surface adsorption and cementation).

Further corroboration was provided by atomic force microscopy (AFM) in Section 2.3, where surface adhesion forces for the three systems were measured as 26.6 nN, 38.6 nN, and 106 nN, respectively (Figure 7c,f,i). The enhancement is attributed to the effective adsorption of ANDT-70 onto bentonite slurry lamellae and its ability to crosslink and aggregate them, thereby improving the overall cohesion of the system.

In summary, results from Raman spectroscopy/mapping and AFM collectively confirm that microgel ANDT-70 significantly improves the surface cementation and adhesion capabilities of bentonite slurry systems.

## 3. Conclusions

This study successfully prepared the core–shell-structured microgel ANDT-70, designed specifically for high-temperature and high-salinity environments, with the systematic evaluation of its thermal stability, salt resistance, and bonding adhesion capabilities. The results show that ANDT-70 maintains excellent structural stability at 260 °C, with minimal changes in viscosity after long-term thermal aging. Compared with representative salt-tolerant materials reported in the literature, ANDT-70 demonstrates stronger tolerance to salt ions under harsh conditions.

AFM analysis confirms that ANDT-70 improves the dispersion of bentonite slurry and reduces the risk of salt-induced degradation. Structural characterization using SEM, TEM, Zeta potential analysis, and Raman mapping shows that ANDT-70 can stably adsorb onto the surface of bentonite lamellae. This adsorption enhances particle interaction through electrostatic forces and hydrogen bonding, forming a stable cementation network. The presence of this network significantly improves the cohesion and stability of bentonite slurry systems in extreme environments. These findings suggest that ANDT-70 is a promising functional microgel for advanced drilling fluids used in complex geological conditions.

## 4. Materials and Methods

### 4.1. Materials

Bentonite, titanium dioxide (TiO_2_), γ-methacryloxypropyltrimethoxysilane (KH-570), 2-acrylamido-2-methylpropane sulfonic acid (AMPS), dimethyl diallyl ammonium chloride (DMDAAC), N-vinyl-2-pyrrolidinone (NVP), and N,N-dimethylacrylamide (DMAA) were all purchased from Shanghai Titan Scientific Co., Ltd. (Shanghai, China). Sodium chloride (NaCl), calcium chloride (CaCl_2_), sodium hydroxide (NaOH), and ethanol were used as standard laboratory reagents.

### 4.2. Synthesis

The experiment was conducted in two main steps: the hydrophobic modification of TiO_2_ and the synthesis of the core–shell-structured microgel ANDT-70. First, TiO_2_ was placed in a drying oven at 50 °C for 24 h and then stored for subsequent use. Subsequently, a 100 mL mixture of absolute ethanol and deionized water was prepared at a mass ratio of 6:4. A predetermined amount of dried TiO_2_ was slowly added into the beaker under magnetic stirring and subjected to ultrasonic treatment for 30 min. The suspension was then transferred to a three-necked flask, and the pH was adjusted to 3–4. A KH-570 solution was slowly added dropwise to the system, followed by continuous stirring in a 60 °C water bath for 24 h to facilitate surface modification. Under ice bath conditions, AMPS was dispersed in deionized water, and NaOH was slowly added to adjust the pH to 7–8. Subsequently, the modified TiO_2_, DMDAAC, DMAA, and NVP were each dispersed in deionized water and then mixed thoroughly. The resulting mixture was transferred into a three-necked flask and allowed to react for 8 h to yield the final microgel product, ANDT-70.

### 4.3. Characterizations

The core–shell-structured microgel ANDT-70 was first dissolved in anhydrous ethanol, followed by multiple washing and precipitation steps to remove impurities and residual moisture. After drying and grinding, the product was thoroughly mixed with potassium bromide and pressed into pellets. The chemical composition and molecular structure were characterized using a Fourier Transform Infrared (FTIR) spectrometer (Model IS10, Nicolet, WI, USA) over the wavenumber range of 4000–400 cm^−1^. To further evaluate the thermal stability of ANDT-70, a sample of 20–30 mg was weighed into a crucible and subjected to thermogravimetric analysis (TGA) using a STA 449 F3 thermal analyzer (NETZSCH, Bavaria, Germany) under a nitrogen atmosphere. The gas flow rate was maintained at 50 mL/min, with a heating rate of 10 °C/min, and the maximum test temperature was set at 600 °C.

### 4.4. Thermal, Salinity, and Long-Term Aging Tests

Experimental procedures were as follows: (1) A specified amount of deionized water was added to a high-speed stirring cup, followed by the gradual addition of 4 wt% bentonite and 0.2 wt% anhydrous sodium carbonate under continuous stirring. The mixture was stirred for 1 h and then allowed to stand for 24 h to obtain a bentonite slurry. (2) The microgel ANDT-70 and salt ions were added sequentially to the prepared bentonite slurry, and the mixture was stirred for 30 min to ensure uniform dispersion. (3) The resulting mixture was subjected to thermal aging in a high-temperature roller oven (Model MK-GRL 4, Hengtaida Instruments, Qingdao, China) at 200 °C, 220 °C, 240 °C, and 260 °C for 16 h, 48 h, 64 h, and 72 h, respectively. (4) The rheological properties of the aged samples were measured using a six-speed rotational viscometer (Model ZNN-D 6, Hengtaida Instruments, Qingdao, China). To ensure the reliability of the experimental results, an uncertainty analysis was conducted. The measurement uncertainties primarily originated from instrument precision, sample preparation, and repeated operations. Each set of experiments was performed at least three times, and the average values were used for analysis. The results indicated that these uncertainties did not significantly affect the overall observed trends, thereby further confirming the robustness of the study’s conclusions.

### 4.5. Bonding Adhesion Test

The microgel ANDT-70 system was dispersed in an appropriate mixture of deionized water and ethanol. After 3 min of ultrasonication, the resulting suspension was diluted to a concentration of 0.1–0.5 mg/mL to ensure optimal signal intensity during measurement. The well-dispersed solution was then carefully transferred using a pipette to the Zetasizer Nano ZS90 (Malvern Instruments, Worcestershire, UK) for Zeta potential measurement. To ensure the reliability of the experimental results, an uncertainty analysis was conducted. The measurement uncertainties primarily originated from instrument precision, sample preparation, and repeated operations. Each set of experiments was performed at least three times, and the average values were used for analysis. The results indicated that these uncertainties did not significantly affect the overall observed trends, thereby further confirming the robustness of the study’s conclusions.

## Data Availability

The original contributions presented in this study are included in the article. Further inquiries can be directed to the corresponding author.
